# Study of Transcriptional Effects in *Cis* at the *IFIH1* Locus

**DOI:** 10.1371/journal.pone.0011564

**Published:** 2010-07-13

**Authors:** Hana Zouk, Luc Marchand, Constantin Polychronakos

**Affiliations:** 1 Endocrine Genetics Laboratory, McGill University Health Center, Montreal Children's Hospital Research Institute, McGill University, Montreal, Quebec, Canada; 2 Department of Human Genetics, McGill University, Montreal, Quebec, Canada; 3 Department of Paediatrics, McGill University Health Centre, Montreal, Quebec, Canada; Mayo Clinic College of Medicine, United States of America

## Abstract

**Background:**

The Thr allele at the non-synonymous single-nucleotide polymorphism (nsSNP) Thr946Ala in the *IFIH1* gene confers risk for Type 1 diabetes (T1D). The SNP is embedded in a 236 kb linkage disequilibrium (LD) block that includes four genes: *IFIH1*, *GCA*, *FAP* and *KCNH7*. The absence of common nsSNPs in the other genes makes the *IFIH1* SNP the strongest functional candidate, but it could be merely a marker of association, due to LD with a variant regulating expression levels of *IFIH1* or neighboring genes.

**Methodology/Principal Findings:**

We investigated the effect of the T1D-associated variation on mRNA transcript expression of these genes. Heterozygous mRNA from lymphoblastoid cell lines (LCLs), pancreas and thymus was examined by allelic expression imbalance, to detect effects in *cis* on mRNA expression. Using single-nucleotide primer extension, we found no difference between mRNA transcripts in 9 LCLs, 6 pancreas and 13 thymus samples, suggesting that *GCA* and *FAP* are not involved. On the other hand, *KCNH7* was not expressed at a detectable level in all tissues examined. Moreover, the association of the Thr946Ala SNP with T1D is not due to modulation of *IFIH1* expression in organs involved in the disease, pointing to the *IFIH1* nsSNP as the causal variant.

**Conclusions/Significance:**

The mechanism of the association of the nsSNP with T1D remains to be determined, but does not involve mRNA modulation. It becomes necessary to study differential function of the *IFIH1* protein alleles at Thr946Ala to confirm that it is responsible for the disease association.

## Introduction

Type 1 diabetes (T1D) is a complex disease involving both genetic and environmental factors. This is largely attributed to genetic variation among individuals at several loci. One of them involves the *IFIH1* gene (interferon-induced helicase 1) where the Thr allele at the Thr946Ala polymorphism increases T1D risk [1]. The associated SNP (rs1990760) is embedded in a 236 kb linkage disequilibrium (LD) block on Chr 2q24.3 that includes four genes: *IFIH1*, also known as *helicard* or *MDA-5* (melanoma differentiation associated gene-5); *GCA* (grancalcin); *FAP* (fibroblast activation protein α subunit) and *KCNH7* (potassium voltage gated channel subfamily H7), any of which could harbor a T1D-associated functional variant. *IFIH1* belongs to a family of RNA helicases that bind double-stranded viral RNA [2], [3] and induces a type I interferon anti-viral response [4]. This is particularly interesting given the evidence for a role of enteroviruses in the etiology of T1D [5]–[7]. The recent discovery of rare nsSNPs in *IFIH1* protective of T1D [8], two of which involve loss of function [9], strongly supports it as the gene involved, but locus heterogeneity remains a possibility given that thousands of loci with weak effects likely account for each complex trait [10]. *GCA* encodes a calcium binding protein that is expressed in most immune cells and is associated with degranulation, and consequently, immune reaction [11]. *FAP* encodes a human stromal antigen, which can in turn activate a T-cell mediated cellular response [12]. *KCNH7* encodes a potassium voltage-gated channel which has many roles, most notably, the regulation of insulin secretion [13]. Hence, all genes at the *IFIH1* locus may be interesting potential candidates in the etiology of T1D even though nsSNPs are more likely to have functional effects. Therefore, the *IFIH1* nsSNP obviously remains the strongest functional candidate; however, the fact that it could be merely a marker of association that tags another variant regulating expression levels of *IFIH1* or of neighboring genes, must be ruled out. In the same paper that reported the functional effect of the rare *IFIH1* SNPs, the Thr946Ala nsSNP was not found to have any effect of protein function [9]. However, because a transfection system was used, where *IFIH1* alleles are over-expressed, small differences between them may not be detectable. Allele-dependent specificities for sequence, length, or other characteristics of specific viral RNAs would also not have been detected.

Recently, transcriptional effects at the *IFIH1* locus were reported by Liu et *al.* [14] involving 40% higher expression of the predisposing allele in peripheral blood mononuclear cells (PBMCs) by real-time PCR. Such a large difference in allelic expression ought to be easily detectable in large-scale, whole transcriptome searches of quantitative effects in *cis*. However, in such a genome wide association study of global gene expression in lymphoblastoid cell lines (LCLs) of 400 children using more than 400,000 SNPs, no transcriptional effects *in cis* were observed on the *IFIH1* locus or any of the surrounding regions [15]. In another paper using the same methodology as Liu *et al.* [14], *IFIH1* allelic expression differences were not observed [16]. This inconsistence in reported results could be due to the fact that both groups compared mRNA levels among individuals of different genotypes. This has the disadvantage of introducing a large amount of background noise from different individuals' immune experience, loci in trans [17], quality of RNA extraction (including mRNA degradation), assay variance, loading normalization, etc., making it difficult to properly detect the typically small functional effects seen at complex-trait loci. A much more robust approach to measure mRNA variation, known as allelic expression imbalance (AI), removes this noise by comparing expression levels of the two alleles originating from the same individual in samples heterozygous for a transcribed SNP. This method has been well validated in our laboratory [18], [19], and others [17], [20]–[23].

AI could stem from allele-dependent effects of one or more polymorphisms in *cis* that alter promoter function as well as that of other regulatory elements such as enhancers and silencers, potentially affecting transcription or RNA stability. Thus, the presence of a regulatory polymorphism that is in LD with the marker SNP could result in AI [21].

In AI assays, each allele acts as an internal control for confounding factors that alter the overall expression of the gene in question, thus maximizing the sensitivity of detecting effects in *cis* on mRNA expression in the same RNA sample, from the same individual. In samples that are heterozygous for a *cis*-acting regulatory variant, one allele will be expressed at a higher level than the other [21], [24], [25]. Heterozygous genomic DNA from the same source is used as a control for 1∶1 stoichiometry [24]. It is worth noting that another advantage of AI is that this assay relies on the comparison of allelic ratio in DNA and mRNA of each individual. Thus, it automatically controls for any polymorphisms present in the primer sites or copy number variation encompassing the gene studied, which normally exert a similar effect on both DNA and mRNA [26].

The purpose of this study was to investigate the effect of the T1D-associated variation on mRNA expression of *IFIH1* and all other genes in the LD block by allelic expression imbalance, using single-nucleotide primer extension (SNuPE) on RT-PCR products of heterozygous lymphocyte, thymic and pancreatic RNA samples, to cover tissues most important in T1D.

## Materials and Methods

### SNP selection

The T1D-associated SNP, rs1990760 (T946A), was selected to assess its effect on *IFIH1* expression levels. An intronic SNP was selected for each of the other 3 genes since there are no suitable common exonic SNPs. Intronic SNPs have been shown to yield similar allelic expression levels to those obtained using exonic SNPs, provided that the genes are highly expressed in the tissue sample, and that the unspliced mRNA (or heteronuclear RNA [hnRNA]) can be successfully amplified and detected [26]. The three selected intronic SNPs were in high LD with each other and with the rs1990760 SNP (r^2^ = 0.513–0.739) (Table 1), and had higher minor allele frequencies (MAF = 0.398–0.492) than the T1D-associated SNP (MAF = 0.392), in order to maximize the number of heterozygotes obtained.

**Table 1:** Pairwise Linkage Disequilibrium Coefficients of SNPs at the *IFIH1 locus*.

SNPs	rs1990760 (IFIH1)	rs7587426 (GCA)	rs2075302 (FAP)	rs2068330 (KCNH7)
rs1990760 (IFIH1)	-	0.700	0.513	0.738
rs7587426 (GCA)	**0.959**	-	0.586	0.643
rs2075302 (FAP)	**0.908**	**0.846**	-	0.370
rs2068330 (KCNH7)	**0.859**	**0.919**	**0.770**	**-**

Numbers in bold represent D′ values (lower diagonal), upper diagonal represent R^2^ values.

### Samples

Lymphoblastoid cell lines (LCLs) from the CEU collection were used. These were unrelated individuals residing in Utah with ancestry from western and northern Europe genotyped for millions of SNPs genome-wide, as part of the HapMap project. LCL samples heterozygous for all of the chosen SNPs were grown in RPMI 1640 medium (Gibco, CA, USA), supplemented with penicillin/streptomycin, 2mM L-glutamine, non-essential amino acids, and 15% heat-inactivated fetal bovine serum (Multicell, RI, USA). Cells were pelleted when they reached a density of about 1.0×10^6^ cells/ml. Thymic and pancreatic samples were obtained from our collection of frozen fetal tissues as previously described [18], [27]. Written informed consent was obtained from all individuals included in this study and was approved by the Research Ethics Board of the hospitals where the recruitments took place: for LCLs, under the auspices of the Centre de L' 'étude du Polymorphisme Humain, Paris, France; for thymic and pancreatic samples, by the Royal Victoria Hospital Research Ethics Board (McGill University Health Centre), Montréal, Québec, Canada.

### DNA and RNA extraction

Extraction of nucleic acids from pancreatic and thymic tissues has been described elsewhere [28], and RNA integrity was assessed by the 2100 Bioanalyzer (Agilent, CA, USA). LCL genomic DNA was extracted using the QIAamp DNA Mini Kit (Qiagen, Germany) and RNA was isolated using the RNeasy Plus Mini Kit (Qiagen, Germany) following the manufacturer's protocol.

### cDNA synthesis and PCR

In a typical reaction, 2.5 µg aliquots of total RNA were treated with 1 U of DNAse I for 30 minutes at 37°C following the manufacturer's protocol (Ambion, TX, USA). Reverse transcription (RT) was carried out under standard conditions using random hexamer primers (Invitrogen, CA, USA) and 1000 ng of total unfragmented RNA, or RNA that has been subjected to chemical fragmentation according to the manufacturer's protocol (Ambion, TX, USA), along with SuperScript II reverse transcriptase according to the manufacturer's instructions (Invitrogen, CA, USA). RNA was also primed with oligo-dt primers. No detectable levels of RT-PCR product were observed in RNA samples if reverse transcriptase was omitted. PCR amplification for genomic DNA and cDNA samples for *IFIH1*, (along with a minus-RT control) was carried out using primers that amplify exon 15 and thus are capable of amplifying both DNA and cDNA. All other genes were studied using primers located in intronic regions, thus amplifying hnRNA and DNA. Primer and probe sequences are listed in Table S1. To ensure uniform conditions, each cDNA sample was assayed with its corresponding heterozygous genomic DNA.

Briefly, 40 ng of DNA or cDNA were combined with 10 µM of each primer pair, 10 mM dNTPs, 50 mM MgCl_2_, 1× PCR Buffer, and 0.3 U of *Taq* Polymerase (Invitrogen, CA, USA), in a total volume of 25 µL. Each PCR reaction consisted of an initial denaturation step at 94°C for 5 min, followed by 35 cycles of denaturation at 94°C for 30 s; annealing at 49.5°C for *IFIH1* for 30 s and extension at 72°C for 30 s, as well as a final extension step of 7 min. *GCA*, *FAP* and *KCNH7* were amplified using the same conditions as described above, with an annealing temperature of 56.5°C. PCR products were subjected to electrophoresis in a 1.5% agarose gel. All samples were run in parallel for each gene in each tissue type.

### Single nucleotide primer extension (SNuPE)

In order to identify heterozygotes for the chosen marker polymorphisms, 38 thymic and 23 pancreatic samples were initially genotyped using single nucleotide primer extension with dideoxy-NTPs (ddNTPs) labeled with different fluorochromes corresponding to each allele [23], [29], [30]. Briefly, the PCR amplicons were extracted and purified from agarose gels using columns from the QIAquick Gel Extraction Kit (Qiagen, Germany), following the manufacturer's protocol. Primer extension was then carried out by combining 2 µL of the purified PCR product with 5 µL of the ABI Prism SNaPshot Multiplex Kit (Applied Biosystems, CA, USA), 2.5 µM of the appropriate extension probe, and 3 µL of water, in a total volume of 10 µL. Primer extension thermocycling conditions consisted of an initial step of 95°C for 2 minutes, followed by 25 cycles of 95°C for 10s, and 60°C for 35 s for *IFIH1*. For the other genes, the denaturation step of 95°C for 10s was followed by cycling at 50°C for 5s then 60°C for 30s. Following primer extension, the products were treated with 1 U of shrimp alkaline phosphatase (Roche, IN, USA) to remove unincorporated ddNTPs, for 1 hr at 37°C, and then the enzyme was deactivated for 15 min at 75°C. Aliquots of 1 µL of SnaPshot product and 9 µL of Hi-Di formamide were loaded into a 3100 DNA sequencer (Applied Biosystems, CA,USA). Products were electrophoresed on a 36-cm capillary array at 60°C. As with cDNA synthesis and PCR steps, all samples were run simultaneously for each gene in each tissue type. Data was processed using Genescan Analysis version 3.7 software (Applied Biosystems, CA,USA). Peak heights representing allele-specific extended primers were calculated using GeneScan in order to generate a ratio of allelic representation. The area under the curve of the peak representing a particular allele is proportional to the abundance of that allele in the sample. Once heterozygous samples were identified, they were re-run simultaneously with their corresponding cDNA to evaluate the relative abundance of the two alleles at a particular polymorphism, using the same protocol. Hence, this enabled us to use the PCR product of the same sequence from genomic DNA as a control for 1∶1 stoichiometry, or a 50∶50 allele ratio. For reproducibility purposes, all samples showing a relative allele difference greater than 40% than the genomic average would be retested two more times, in two separate RT-PCR reactions, along with the corresponding genomic DNA. This was not necessary, as all allele ratios fell within the 40% range.

### Statistical Analysis

The ratio of one allele over the other for each SNP was calculated for the AI assay. The ratio for each sample was divided by the average genomic ratio for that assay batch in order to account for differences in probe and fluorochrome efficiencies. Peak height ratios corresponding to each allele in individual DNA samples ranged from 0.79 to 1.21 for *IFIH1*, from 0.80 to 1.35 for *GCA*, and from 0.93 to 1.06 for *FAP* SNP. Allelic expression differences between DNA and cDNA were evaluated by the student t test for statistical significance. A two-tailed level of 0.05 was chosen for a type I error rate. Power analysis was calculated for detection of a 40% difference in relative expression of the two alleles, at α = 0.01 (similar to Liu *et al.* [14]).

## Results

Our working sample was comprised of 9 LCL, 13 thymus, and 6 pancreas samples that were found to be heterozygous for all four SNPs. *IFIH1* and *GCA* gene expression was detected in all LCL, thymus and pancreas samples. *FAP* was exclusively expressed in pancreas and thymus, but not LCLs. We were not able to detect *KCNH7* expression in any of the three tissues.

**Figure 1:**
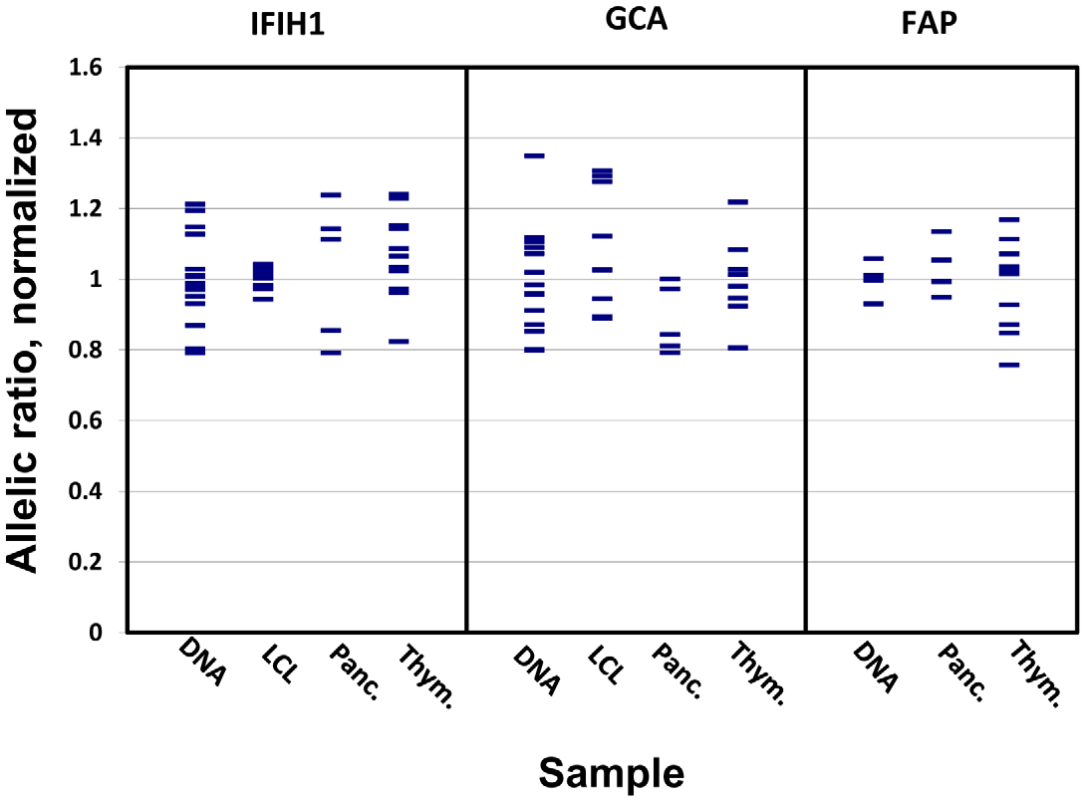
Allelic ratio distribution at the *IFIH1* locus. 9 Lymphoblastoid cell lines (LCL), 6 pancreas (Panc.) and 13 thymus (Thym.) tissue from individuals heterozygous for the selected marker SNPs for each gene were used to assess allelic imbalance at the *IFIH1* locus. Relative allelic abundance in individual samples has been normalized to the mean genomic DNA ratio (equal to1) and normalized sample RNA ratios were compared to those of normalized genomic DNA for each gene in each tissue. The average means ± SEM are summarized in table 2, along with the statistical analysis. Our power to detect a difference of 40% in the means of DNA and LCL RNA was >99%.

The calculated allelic ratios for each SNP representing each gene in the *IFIH1* locus and their distribution in the different tissues that were assayed are shown in Table 2 and Figure 1 respectively. The allelic ratio distribution of *IFIH1*, *GCA*, and *FAP* cDNA do not significantly differ from that of their corresponding DNA. After correction by the genomic DNA allele proportion, the average ratio (mean ± SEM) of the major allele (T) over the minor allele (C) of the rs1990760 SNP in *IFIH1* in LCLs is 1.0029±0.0106, *p* = 0.8477 (Table 2), indicating the absence of an AI effect due to a common genetic variation at the *IFIH1* gene in LCLs. The same is observed in pancreas and thymus. No evidence of significant transcriptional effect was seen in any of the other genes in all the assayed tissues and cells (Table 2).Our approach had a 99% power to detect a transcriptional effect of rs1990760 on *IFIH1* in LCLs, of the magnitude reported by Liu *et al.* [14] (40% allelic difference at α = 0.01). We also have 99% power to detect a 25% difference in expression between the two *IFIH1*alleles. Since all RNA samples were DNAse-treated prior to RT-PCR and did not generate detectable PCR product in the absence of an RT step, it is highly improbable that our results were influenced in some way by genomic DNA contamination. It has been recently suggested [31] that differential secondary structure of RNA alleles may interfere with quantitative comparisons through a differential effect on the efficiency of reverse transcription, creating spurious allelic imbalance or conceivably masking true imbalance (if it happens to be exactly equal and in the opposite direction). To deal with this, minimization of secondary structure by fragmenting the RNA prior to reverse transcription (RT) was recommended. To see whether this may be a problem in the specific case of *IFIH1*, we compared allelic ratios obtained with or without fragmentation of the RNA prior to RT. In twelve independent comparisons, the mean allelic ratio (normalized for the average DNA ratio) was 0.95±0.07 (SEM) for unfragmented vs. 1.00±0.05 for fragmented RNA (p = 0.56, 99% power to detect a 40% effect at α = 0.01, 86.7% power to detect a 25% effect at α = 0.05). Oligo-dT priming of the RT, suggested as an alternative, also gave nearly-identical results (0.98±0.05 [SEM]). We, therefore, concluded that interference by secondary structure was not an issue in allelic *IFIH1* measurements.

**Table 2:** Summary of difference in allelic variation at the *IFIH1* locus.

Gene	SNP	Alleles	Tissue	Mean allelic ratio DNA ± SEM^a^	Mean allelic ratio cDNA ± SEM^a^	p^b^	Power^c^
IFIH1	rs1990760	T/C	LCL	1.0000±0.0077	1.0029±0.0106	0.8477	100.0%
IFIH1	rs1990760	T/C	Thymus	1.0000±0.0596	1.0855±0.0341	0.2002	99.9%
IFIH1	rs1990760	T/C	Pancreas	1.0000±0.1009	1.0630±0.0791	0.6334	70.7%
GCA	rs7587426	C/T	LCL	1.0000±0.0415	1.0939±0.0637	0.2373	99.6%
GCA	rs7587426	C/T	Thymus	1.0000±0.0435	0.9999±0.0427	0.9994	100.0%
GCA	rs7587426	C/T	Pancreas	1.0000±0.1751	0.8840±0.0429	0.4393	36.1%
FAP	rs2075302	T/C	LCL	could not be detected by PCR, not expressed in B lymphocytes		/	/
FAP	rs2075302	T/C	Thymus	1.0000±0.0375	0.9835±0.0408	0.8387	100.0%
FAP	rs2075302	T/C	Pancreas	1.0000±0.0030	1.0251±0.0321	0.6606	100.0%
KCNH7	rs2068330	C/G	LCL	could not be detected by PCR, not expressed in B lymphocytes		/	/
KCNH7	rs2068330	C/G	Thymus	could not be detected by PCR, not expressed in thymus		/	/
KCNH7	rs2068330	C/G	Pancreas	could not be detected by PCR, not expressed in pancreas		/	/

a n = 9 for LCLs, n = 13 for thymus, n = 6 for pancreas.

b statistical significance as measured by the two-tailed student *t* test.

c statistical power to detect a 40% difference of expression between alleles, at an α = 0.01.

## Discussion

The association of the *IFIH1* locus with T1D is supported by a large number of genetic studies [1], [32], [33]. Yet, the presence of strong LD in the block has made it far more challenging to identify the disease-causing polymorphism or haplotype. Thus, detailed functional analysis is required. A recent study that showed higher *IFIH1* mRNA levels in PBMCs of individuals with the susceptible genotype of the T1D associated SNP by real-time RT-PCR suggested that a differential regulation of *IFIH1* expression could be, at least in part, responsible for the T1D risk [14], while another study was not able to show any difference in *IFIH1* allelic expression using the same technology [16]. This approach, comparing *IFIH1* levels across individuals with different genotypes [14], [16], introduces substantial noise from inter-individual variability and by factors such as assay and batch variance, *trans*-acting genetic factors, immune experience, cell proportions in the PBMC mixtures and the presence of polymorphisms in other genes that may alter the expression level in *trans* [23], [34], thus diluting the effect of *cis*-acting influences in expression studies [35]. In order to resolve this controversy, we used SNuPE, a method that relies on the comparison of alleles within rather than between samples, which removes external confounding factors. The SNuPE technique is quite accurate and thus allows small differences in allelic ratios to be reliably detected [24]. We have previously validated the accuracy of this method by showing an excellent correlation between observed and expected allelic ratios [19].

The concern that allelic expression in transformed cultured cell lines may not accurately represent what occurs in human tissues was addressed in a recent paper that explored whether different culture conditions (passage number, pH, nutrient concentration, cell density, etc.) influence AI results [26]. It was found that estimations of AI after different passages were not significantly different from one another. Another potential limitation of our study was our use of total pancreas rather than islets. Since AI of some genes may be tissue specific, we may have missed an islet-specific effect. This, however, seems unlikely since both endocrine and exocrine pancreatic tissues are of similar origins, and likely exhibit similar allelic expression.

We were unable to replicate the results reported by Liu *et al.* [14], using a much more sensitive, accurate and reproducible method that has the power to detect an effect that is similar to that was reported. This is in accordance with the expectation that the nsSNP is the most likely functional candidate.

In summary, our results suggest that *GCA* and *FAP* are not involved in T1D since we observed no AI and there are no nsSNPs of high enough frequency to explain the effect [1]. This reinforces the role of the *IFIH1* nsSNP as a potential causal variant. In addition, *KCNH7* was not expressed in LCLs, fetal pancreas or thymus, and thus could not be assayed for AI. Therefore, a transcriptional effect of the Thr946Ala SNP, or any variant in LD with it, on *KCNH7* cannot be ruled out.

While our study shows no transcriptional effects of the Thr946Ala SNP or the other chosen variants that were in tight LD with it on the three assayed genes, we cannot exclude the possibility of the presence of other *cis*-regulatory variants with a lower minor allele frequency exerting transcriptional effects on these genes, and being detected by our AI assay. While in some samples, the cDNA allelic ratio deviates by at least 20% from the genomic ratio (Figure 1), we are unable to conclude whether we are observing transcriptional effects of such a polymorphism in *cis*, or whether these merely reflect measurement error. Nonetheless, the *IFIH1* gene has been sequenced extensively, and no rare variants have been found that can explain the association of the common Thr946Ala SNP to T1D [8].

Thus, the mechanism of the observed association of the rs1990760 with T1D remains to be determined, but does not involve modulation of mRNA. Although the *IFIH1* nsSNP, rs1990760 (Thr946Ala substitution), does not reside in either the signaling CARD domain or RNA binding helicase domain of the protein, this region of the protein is conserved between mammals and may have other, unknown functions or have an effect on the active domains through changes in tertiary structure [1], which may well be affected by the Thr946Ala SNP. This is a non-conservative substitution, changing the polarity of the amino acid from polar to non-polar. Recently, four rare mutations have been identified in *IFIH1*, each of which separately lowered the risk of developing T1D [8], independently of the effect of the Thr946Ala SNP. Two of these four variants have been shown to be loss of function mutations, dramatically reducing *IFIH1* protein activity or its RNA binding ability [9]. That loss of *IFIH1* function protects from T1D would indicate that the risk allele is related to an exaggerated immune response rather than imperfect anti-viral defense. By implication, if Thr946Ala itself is indeed the functional variant responsible for its T1D association, one would expect the diabetes-predisposing Ala allele to represent gain of function even though it was derived by mutation of the conserved ancestral Thr allele. However, such a mutation needs not to result in diminished protein function, as conservation is driven by fitness of the organism, not higher level of protein function. Such an example can be found in one of the strongest T1D associations, which involves the R620W SNP in *PTPN22* (protein tyrosine phosphatase, non-receptor type 22), where the 620W disease-associated variant, derived in an even more conserved context, is a gain-of-function variant, with increased catalytic activity [36]. In the same paper which showed that two of the four rare *IFIH1* variants are loss of function mutations, the Thr946Ala nsSNP was not found to affect dsRNA binding or consequent IFN gene activation in mouse embryonic fibroblast cells in culture [9]. However, in a transfection system, an excess of *IFIH1*, expressed above the threshold where small allelic differences in dsRNA binding affinity and/or IFN response can be detected, subtle effects could have been missed. It may also be that the SNP alters interaction with specific dsRNA structures not modeled by the mimic used. Alternatively, the Thr946Ala nsSNP could affect translational efficiency or may even be involved in the post-translational processing of the *IFIH1* protein. Additional work on the Thr946Ala SNP is therefore necessary to discover how it alters *IFIH1* function and gain insight on how it affects T1D pathogenesis.

## Supporting Information

Table S1 Primer sequences and probes for each SNP
(0.02 MB XLS)
